# Transcriptomic difference in bovine blastocysts following vitrification and slow freezing at morula stage

**DOI:** 10.1371/journal.pone.0187268

**Published:** 2017-11-02

**Authors:** Alisha Gupta, Jaswant Singh, Isabelle Dufort, Claude Robert, Fernanda Caminha Faustino Dias, Muhammad Anzar

**Affiliations:** 1 Agriculture and Agri-food, Saskatoon Research and Development Center, Saskatoon, Saskatchewan, Canada; 2 Department of Veterinary Biomedical Sciences, Western College of Veterinary Medicine, University of Saskatchewan, Saskatoon, Saskatchewan, Canada; 3 Centre de recherche en biologie de la reproduction, Faculté des sciences de l'agriculture et del'alimentation Pavillon INAF, local 2742 Université Laval, Québec, Québec, Canada; Justus Liebig Universitat Giessen, GERMANY

## Abstract

Cryopreservation is known for its marked deleterious effects on embryonic health. Bovine compact morulae were vitrified or slow-frozen, and post-warm morulae were cultured to the expanded blastocyst stage. Blastocysts developed from vitrified and slow-frozen morulae were subjected to microarray analysis and compared with blastocysts developed from unfrozen control morulae for differential gene expression. Morula to blastocyst conversion rate was higher (*P* < 0.05) in control (72%) and vitrified (77%) than in slow-frozen (34%) morulae. Total 20 genes were upregulated and 44 genes were downregulated in blastocysts developed from vitrified morulae (fold change ≥ ± 2, *P* < 0.05) in comparison with blastocysts developed from control morulae. In blastocysts developed from slow-frozen morulae, 102 genes were upregulated and 63 genes were downregulated (fold change ≥ ± 1.5, *P* < 0.05). Blastocysts developed from vitrified morulae exhibited significant changes in gene expression mainly involving embryo implantation (*PTGS2*, *CALB1*), lipid peroxidation and reactive oxygen species generation (*HSD3B1*, *AKR1B1*, *APOA1*) and cell differentiation (*KRT19*, *CLDN23*). However, blastocysts developed from slow-frozen morulae showed changes in the expression of genes related to cell signaling (*SPP1*), cell structure and differentiation (*DCLK2*, *JAM2 and VIM*), and lipid metabolism (*PLA2R1 and SMPD3*). In silico comparison between blastocysts developed form vitrified and slow-frozen morulae revealed similar changes in gene expression as between blastocysts developed from vitrified and control morulae. In conclusion, blastocysts developed form vitrified morulae demonstrated better post-warming survival than blastocysts developed from slow-frozen morulae but their gene expression related to lipid metabolism, steroidogenesis, cell differentiation and placentation changed significantly (≥ 2 fold). Slow freezing method killed more morulae than vitrification but those which survived up to blastocyst stage did not express ≥ 2 fold change in their gene expression as compared with blastocysts from control morulae.

## Introduction

Cryopreservation of bovine embryos is widely used for trade of genetically superior animals and conservation of genetic diversity. Bovine embryos are commonly frozen with either slow freezing or vitrification method [1]. Both techniques differ in concentration of cryoprotectants and cooling rates [2]. Slow freezing is currently a gold standard method for cryopreservation of bovine embryos. However, it damages mammalian embryos due to intracellular ice formation and toxic effect of the permeating cryoprotectants (~1–2 M). The intracellular ice formation is dependent on cooling rate and surface area/volume of cells [3]. Cryopreservation leaves deleterious effects on oocytes and embryos at developmental, morphological and biochemical levels [4,5]. Over the years, the attempts have been made to minimize damage to morulae/blastocysts caused by cryoprotectant-associated toxicity and intracellular ice formation during cryopreservation [1,6]. Vitrification includes the use of highly viscous solution of cryoprotectants (~7–8 M) to achieve a glass-like state with ultra-rapid cooling rates (>5000°C/min) avoiding the intracellular ice formation [7,8]. Following vitrification, bovine embryos undergo extra- and intra-cellular glass-like state instead of ice crystal formation [9,10]. The vitrified bovine embryos have improved or equivalent survival [11], blastocyst hatching and pregnancy rates [10,12] as compared with slow-frozen embryos. It is anticipated that vitrification will gradually replace slow freezing for embryo cryopreservation [2]. Vitrification may be a suitable cryopreservation method for in vitro produced embryos which do not survive very well following slow freezing method, compared to in vivo produced embryos [13]. Both, vitrification and slow freezing cause intracellular/extracellular fractures in freezing planes, acute shrinkage in cell volume and organelle damage in mammalian embryos [1,14,15].

The gene expression in bovine oocytes at different development stages and in pre-implantation embryos has been studied [16–18]. The gene expression in oocytes, granulosa cells and pre-implantation embryos changed during extreme stress conditions such as heat shock [19] and hormonal imbalance [20–22]. Vitrification of in vitro produced bovine blastocysts up-regulated genes involved in stress response [23]. Vitrification of mouse embryos exhibited differential apoptotic and DNA methylation gene expression [24]. TUNEL-based assays on embryos showed low DNA-integrity indices after slow freezing and vitrification in mouse, human and bovine species [25,26]. Vitrification skewed inner cell mass to trophoblast ratio and generated reactive oxygen species in mouse embryos [27]. On contrary, slow-frozen bovine embryos exhibited higher pro-apoptotic gene expression compared to vitrified embryos [28]. These studies have examined individual genes in isolation. In order to fully understand the effects of vitrification and slow freezing on cellular and molecular pathways, there is a need to compare the global gene expression in *in vitro* produced bovine embryos. Such cryopreservation-related subtle but cumulative changes may influence the embryo development at a morphological level and may have long-term effects. The objectives of this study were to examine blastocyst development in vitrified, slow-frozen and unfrozen control bovine morulae, and to investigate their differential gene expression, using microarray analysis.

## Material and methods

### Chemicals and culture media

All chemicals were purchase from Sigma-Aldrich^®^ (Oakville, ON, Canada), unless otherwise specified. Calf serum (CS; Cat#12484–010), Dulbecco’s Phosphate Buffer Saline (DPBS Ca^2+^-Mg^2+^ plus; Cat# 21300–025) and Tissue Culture Medium-199 (TCM-199 (Cat# 12340–030) were purchased from Invitrogen Inc. (Burlington, ON, Canada). Lutropin-V (LH; Cat # 1215094) and Folltropin-V (FSH; Cat # PHD075) were obtained from Bioniche^®^ Animal Health, Inc. (Belleville, ON, Canada). Cryotops for vitrification and 0.25-ml straws for slow freezing were purchased from Kitazato^®^ Co. (Fuzi, Shizuoka, Japan) and IMV^®^ Tech. (Woodstock, ON, Canada), respectively.

### Cumulus oocyte complex (COC) collection

Cow ovaries were collected from a commercial slaughterhouse (Cargill^®^, Calgary) and transported to Saskatoon at 20–25°C within 12–18 h. Ovaries, after trimming extra tissue, were washed with normal saline at room temperature. Follicular fluid containing cumulus oocyte complexes (COCs) was aspirated from <4mm ovarian follicles using an 18-gauge needle attached to 5-ml syringe, and pooled among ovaries for further processing.

### In vitro embryo (morulae) production

The pooled follicular fluid was searched for COCs under stereomicroscope. COCs were washed in holding solution (HS; 5% CS in 1X DPBS) and graded as described earlier [29]. First and second grade COCs were washed (3X) in maturation medium [TCM-199 supplemented with 5% CS, LH (5 μg/ml), FSH (0.5 μg/ml) and gentamicin (0.05 μg/ml)]. For *in vitro* maturation, groups of ~20 oocytes were placed in 100 μl droplets of maturation medium under mineral oil, and incubated at 38.5°C, 5% CO_2_ in air and saturated humidity, for 22–24 h.

For *in vitro* fertilization (IVF), two semen straws from a fertile bull were thawed at 37°C for 1 min. Semen was pooled and washed through Percoll gradient (45% and 90%) [30]. After washing, sperm were diluted in Brackett-Oliphant (BO) fertilization medium to a final concentration 3x10^6^/ml [31] [BO stock A + BO stock B + sodium pyruvate (1.3% w/v) + gentamicin (0.05 μg/ml)]. Following IVM, groups of 20 mature COCs were washed (3X) in BO medium supplemented with 10% (w/v) bovine serum albumin and added to 100 μl droplets of sperm in BO medium, under mineral oil, and incubated at 38.5°C, 5% CO_2_ in air and saturated humidity. After 18–22 h co-incubation of sperm and COCs, zygotes were washed and cultured *in vitro* (IVC) in CR1aa medium [32] supplemented with 5% (v/v) CS at 38.5°C, 5% CO_2_, 5% O_2_ and 90% N_2_ in air, and saturated humidity. On d7 post-IVF, compact morulae were collected, washed in HS and randomly divided in control, vitrification or slow freezing groups. Control morulae were incubated in IVC medium for 24–48 h. The remaining morulae underwent cryopreservation (vitrification or slow freezing) as follows.

### Cryopreservation of morulae

#### Vitrification

Vitrification was conducted as described earlier [33]. Briefly, morulae were washed in HS and equilibrated in vitrification solution 1 [VS1; 7.5% Ethylene glycol (EG, v/v) + 7.5% dimethyl sulfoxide (DMSO, v/v) + 20% CS (v/v) in 1X DPBS] for 5 min at room temperature. Morulae (n = 3 to 4 in a given batch) were passed through three 20-μl droplets of vitrification solution 2 [VS2; 15% EG + 15% DMSO + 20% CS + 17.1% sucrose (w/v) in 1X DPBS] at 37°C within 1 min, placed on cryotop (Kitazato^®^ Co., Fuzi, Shizuoka, Japan) in individual droplet with minimal volume of VS2, immediately plunged in liquid N_2_ and stored for at least 24 h before warming.

#### Slow freezing

Slow freezing was done as described earlier [34]. Briefly, morulae were washed (1X) and incubated in cryoprotectant freezing solution [1.5 M glycerol + 5% CS (v/v) in 1X DPBS] for 10 min at room temperature. Morulae were transferred to 0.25-ml plastic straws (IMV^®^ Tech., Woodstock, ON, Canada), sealed and kept in the controlled rate freezer (Bio-Cool^®^ III-80, FTS systems, SP Industries, Inc., Stone Ridge, NY, USA) already set at -7°C, for 5 min. Ice seeding was initiated by touching straws with an ultra-cold Q-tip immersed in liquid N_2_. The straws were placed back in freezer at -7°C for additional 10 min, cooled to -35°C at the rate of 0.5°C/min, quickly plunged in liquid N_2_ and stored for at least 24 h before warming.

#### Warming

Vitrified morulae on cryotop were transferred to warming solution [0.5 M sucrose (w/v) + 20% CS (v/v) in 1X DPBS] at 37°C and incubated for 5 min. Similarly, slow-frozen morulae in 0.25 ml plastic straws were held in air for 10 s and immersed in water bath at 37°C for 1 min. For glycerol removal, slow-frozen morulae were transferred to warming solution [0.7 M sucrose + 5% CS (v/v) in 1X DPBS] at 37°C and incubated for 5 min.

### Morulae culture and blastocyst collection

Post-warm (vitrified and slow-frozen) morulae were washed with HS and cultured in CR1aa medium for 24–48 h to the expanded blastocyst stage. Blastocyst conversion rates (%) were calculated for each treatment group (control, vitrification and slow-freezing) as number of expanded blastocysts out of number of morulae used per group.

Expanded blastocysts (n = 5 to 7 per treatment per replicate) were pooled in 50–100 μl RNAse-free water in 0.5 ml cryovials (RNase-free; Neptune^®^, San Diego, CA). Expanded blastocysts were flash frozen by plunging cryovials in liquid N_2_ and shipped to Dép. des Sciences Animales, Université Laval, Quebec city, QC, for microarray analysis. Total five IVF/IVC/cryopreservation cycles (i.e. biological replicates) were conducted on separate dates. Four biological replicates were used in microarray analysis (i.e. one cryotube per treatment per cycle). Three biological replicates were used in quantitative real-time PCR (qRT-PCR) analysis. Two biological replicates (cycles) were common between microarray analysis and qRT-PCR. Different tubes containing blastocysts (within treatment and replicate) were used for microarray and qRT-PCR analyses.

### Microarray analysis

All procedures for microarray experiment were conducted according to procedures described previously [22,35], with little modifications. Total RNA was extracted from blastocysts developed from vitrified, slow-frozen and control morulae (replicate-wise) using Arcturus Picopure^®^ RNA isolation kit (Cat#KIT0204, Life Technologies, Burlington, ON). The samples were subjected to a DNAse I (Cat#79254, Qiagen^®^ Inc., Toronto, ON) digestion on the column. Total RNA was eluted in 13 μl of elution buffer. The quality and quantity of RNA was analyzed using Agilent 2100 Bioanalyzer^™^ and Agilent RNA 6000^®^ pico kit (Cat# 5067–1513, Agilent technologies, Santa Clara, CA) and stored at -80°C until microarray and qRT-PCR analyses. High quality RNA samples with RNA integrity number (RIN) over 7.0 were amplified using T7 RNA amplification procedure RiboAmp^®^ HS^Plus^ RNA Amplification Kit (Cat# KIT0525, Life^™^ technologies, Burlington, ON) and used for microarray hybridization.

The amplified RNA (aRNA) samples from control, vitrified and slow-frozen blastocysts (replicate wise) were labeled with DY-547/647 (Green–Cy3 and Red–Cy5) fluorescent dyes using Universal Labeling System (ULS^™^) Labeling Kit (Cat# EA-021, Kreatech^®^ Diagnostics, Amsterdam, The Netherlands), as recommended by manufacturer. The unfrozen control group was used as reference for both vitrification and slow freezing groups, therefore 4 μg aRNA from control samples, and 2.5 μg aRNA from each vitrification and slow freezing samples were labeled with Cy3 or Cy5 dyes. Non-reacted residual dyes were filtered out using Picopure RNA isolation kit (Cat#KIT0204, Life^™^ Technologies, Burlington, ON) without DNAse I treatment. Pure labeled aRNA was eluted with 13 μl elution buffer. Labeling efficiency for both dyes was measured with NanoDrop^™^ ND-1000 spectrophotometer (Nanodrop Technologies, Wilmington, DE, USA) with a minimum 30 pmol/μg (dye concentration/aRNA concentration) for each sample.

Two custom-built bovine microarray slides (EmbryoGENE EMBV3 manufactured by Agilent^®^ technologies GEO Accession #: GPL13226, Design ID-028298), were used [36]. Each slide consisted of 4 arrays and each array contained 43,671 probes, including 21,139 unique genes and 9,322 novel transcribed regions (NTRs). One slide was used to compare control (reference) and vitrification groups, and other one to compare control (reference) and slow freezing groups. A hybridization mixture [containing 825 ng of each cyanine (Cy3 or Cy5) labeled aRNA, Agilent spikes, nuclease-free water, 10X blocking agent, and 25X fragmentation buffer], in total volume 55 μl, was pipetted onto the microarray slides. Four biological replicates in each comparison (control vs. vitrified or control vs. slow-frozen) were used in the experimental design, in dye-swap setup. Slides were incubated at 65°C for 17 h, washed with wash buffers, dried and scanned using Tecan PowerScanner^™^ (Tecan Group Ltd., Mannedorf, Switzerland) [36].

The quality of hybridization was determined from the distribution of signals generated by both channels, in addition to the negative and spike-in controls, as reported earlier [36]. Repeatability and specificity of hybridization were visualized through the distribution across channels of repeated and control probes bearing an increasing number of mismatches, respectively. Within an experiment, the set of microarrays was compared through a correlation matrix that enabled the quick identification of poor and divergent replication.

The normalized and differential expression data from FlexArray^®^ were uploaded and analyzed in Ingenuity^®^ Pathway Analysis (IPA) software. Differential gene lists from vitrified vs. control (≥ ± 2-fold change and *P* < 0.05) and slow-frozen vs. control (≥ ± 1.5 fold change, *P* < 0.05), and vitrification vs. slow freezing (≥ ± 2 fold change, *P* < 0.05) were compared for functional analysis to obtain molecular, cellular and functional correlations.

### Quantitative real-time PCR (qRT-PCR) analysis

Total RNA extraction from blastocysts developed from vitrified, slow-frozen and control morulae, and initial processing is described under microarray analysis. Total RNA was reverse transcribed to cDNA using qScript^™^ cDNA supermix (Cat#95048–100, Quanta Biosciences, Inc., MD, USA) following kit instructions. Total cDNA quantity was measured using Nanodrop spectrophotometer and stored in -80°C until further use. In addition, total RNA and cDNA were obtained from extra samples of pooled IVP bovine expanded blastocysts for primer optimization and standard curve generation.

Total 7 genes (*AKR1B1*, *CLDN23*, *CYP11A1*, *KRT19*, *PLAU*, *SPP1 and TKTL1*) and one housekeeping gene (conserved helix-loop-helix ubiquitous kinase, CHUK) [35] were selected for qRT-PCR analysis. Primer testing and optimization was done using end-point PCR implying *Taq* DNA polymerase (Cat#201203, Qiagen) kit. PCR products were visualized on 1% agarose gel, purified, quantified, and sequenced to confirm specificity and validity of primers. The list of selected genes and primers is presented in Table 1.

**Table 1:** Primer sequences used for qRT-PCR.

Gene	Genebank accession no.	Strand (5’-3’)	Primer sequence	Annealing temp (°C)	Product size (bp)
*AKR1B1*	BC110178	Forward	CCAACCACATCGTGCTCTAC	55	163
		Reverse	CCCACCTCGTTCTCATTCTG	55	
*CLDN23*	XM_592516	Forward	AAACACCTGGCTCGGAGTC	55	166
		Reverse	AGGGCCTTGATTCCTCTGG	55	
*CYP11A1*	NM_176644 XM_003587562	Forward	ATCCAGTGTCTCAGGACTTCGT	61	209
		Reverse	GAACATCTTGTAGACGGCATCA	61	
*KRT19*	NM_001015600 XM_592718	Forward	GAGGAGCTGAACAGGGAGGT	61	218
		Reverse	CTGGGCTTCGATACCACTGA	61	
*PLAU*	XM_015460988	Forward	GCTGGTGTTCTGTGTCTG	55	230
		Reverse	GGTCGGAAGGGATAACTG	55	
*SPP1*	NM_174187.2	Forward	ATT GTG GCT TAC GGA CTG	54	196
		Reverse	TTG GCG TGA GTT CTT TGG	54	
*TKTL1*	NM_001003906.1	Forward	ACAAGCCAAGGTGGTCCTGAAGAA	62	185
		Reverse	TAGCACGGGCACTGTCAAGAATGA	62	
*CHUK*	NM_174021.2	Forward	TGATGGAATCTCTGGAACAGCG	56	181
		Reverse	TGCTTACAGCCCAACAACTTGC	56	

The cDNA equivalent to 0.2 embryos was used from each treatment group per replicate. Quantitative real-time PCR was done on Stratagene^®^ Mx3005P fast thermal cycler (Agilent technologies, Santa Clara, CA) using QuantiFast^®^ SYBR^®^ green PCR kit (Qiagen). PCR protocol included initial step at 95°C for 5 min followed by 40 cycles of 95°C for 10 s and 60°C for 1 min.

Cycle threshold (C_T_) values were recorded for each selected gene for every treatment group. These C_T_ values were used to calculate differential expression in blastocysts developed from vitrified and slow-frozen morulae vs. blastocysts developed from control morulae. At first, PCR efficiency was calculated using standard curve data for each gene and software used pair-wise fixed reallocation randomization test using Relative Expression Software Tool (REST^®^ 2009, Qiagen). PCR efficiency ranging between 1.90 and 1.99 (i.e. 90% and 99%) was considered optimum for each gene.

The relative levels of a transcript for each treatment group were calculated as cycle threshold (C_T_) normalized separately (ΔC_T_) for levels of transcripts for house-keeping (CHUK) gene. A lower C_T_ (or Δ C_T_) of “1” indicates approximately a two-fold (2^1^) higher concentration of RNA.

### Statistical analysis

Data on blastocyst development rate were analyzed using link-function for binary distribution (yes/no response variable). Three groups (control, vitrification, slow freezing) were considered categorical fixed-effect explanatory variables and replicate was considered as a random factor. Data were analyzed using Proc Glimmix in SAS^®^ Enterprise Guide 4.3 (SAS). Data were arranged in columns for replicate, treatment, outcome (each embryo was coded as 1 = successful development, 0 = no development). Following common SAS syntax model was used: Proc glimmix method = quad; class = replicate treatment; model outcome (event = “1”) = treatment/ dist = bin link = logit; random intercept/subject = Replicate; run. If the p-value for treatment was ≤0.05, then least square means were compared using Tukey’s test.

Microarray data were normalized and analyzed as described earlier [22]. After laser scanning the slides, image files and median signal intensities from each spot were obtained using Array-Pro^™^ software (Media Cybernetics Inc., Rockville, MD, USA). The gene-spot intensity file was uploaded in MIAME-compliant ELMA (EmbryoGENE Laboratory Information Management System and Microarray Analysis) portal. Data quality control (probe specificity, and variance between biological replicates and between treatments) was monitored by in-built Gydle^™^ software (http://www.gydle.com). The background and spot median intensities were uploaded and analyzed in FlexArray^®^ software Version 1.6.3. For background normalization, background signal intensity was subtracted from median grayscale signal intensity of spots to obtain required correct signal intensity. In case of higher background intensity for a spot than the signal intensity, negative value was replaced with 0.5 as a default (false spots). The median value for each target was transformed to the log_2_ value and normalized “within array” for dye bias using non-parametric regression (locally weighted scatter plot smoothing “lowess”), and subjected to between array normalization to unify intensities across the arrays using quantile normalization methodology (GEO #: GSE45381), respectively [37]. Linear Models for Microarray (LIMMA) and RNA-Seq data simple statistical analyses were done in FlexArray and lists of upregulated and downregulated genes were obtained for each comparison [i.e. vitrification vs. control (reference) group and slow freezing vs. control (reference) group]. Statistically adjusted signal intensity data for vitrification and slow-frozen were derived from the previously acquired data for vitrification-control and slow freezing-control groups and then *in silico* FlexArray analysis was conducted between vitrification and slow freezing (reference) groups. A gene was considered differentially expressed if the fold change was ≥ ± 2 with a *P* < 0.05. As no genes were detected as differentially expressed at fold change ± 2 in the slow freezing vs. control comparison, and additional analysis was performed by lowering the fold change threshold to ≥ ± 1.5. Microarray data have been deposited in NCBI’s Gene Expression Omnibus accessible through GEO SuperSeries accession number GSE95382. The fold changes from microarray and qRT-PCR data were compared using a Student’s t-test in Microsoft^®^ Excel, for each tested gene.

## Results

### Morula to blastocyst development rate

**Fig 1:**
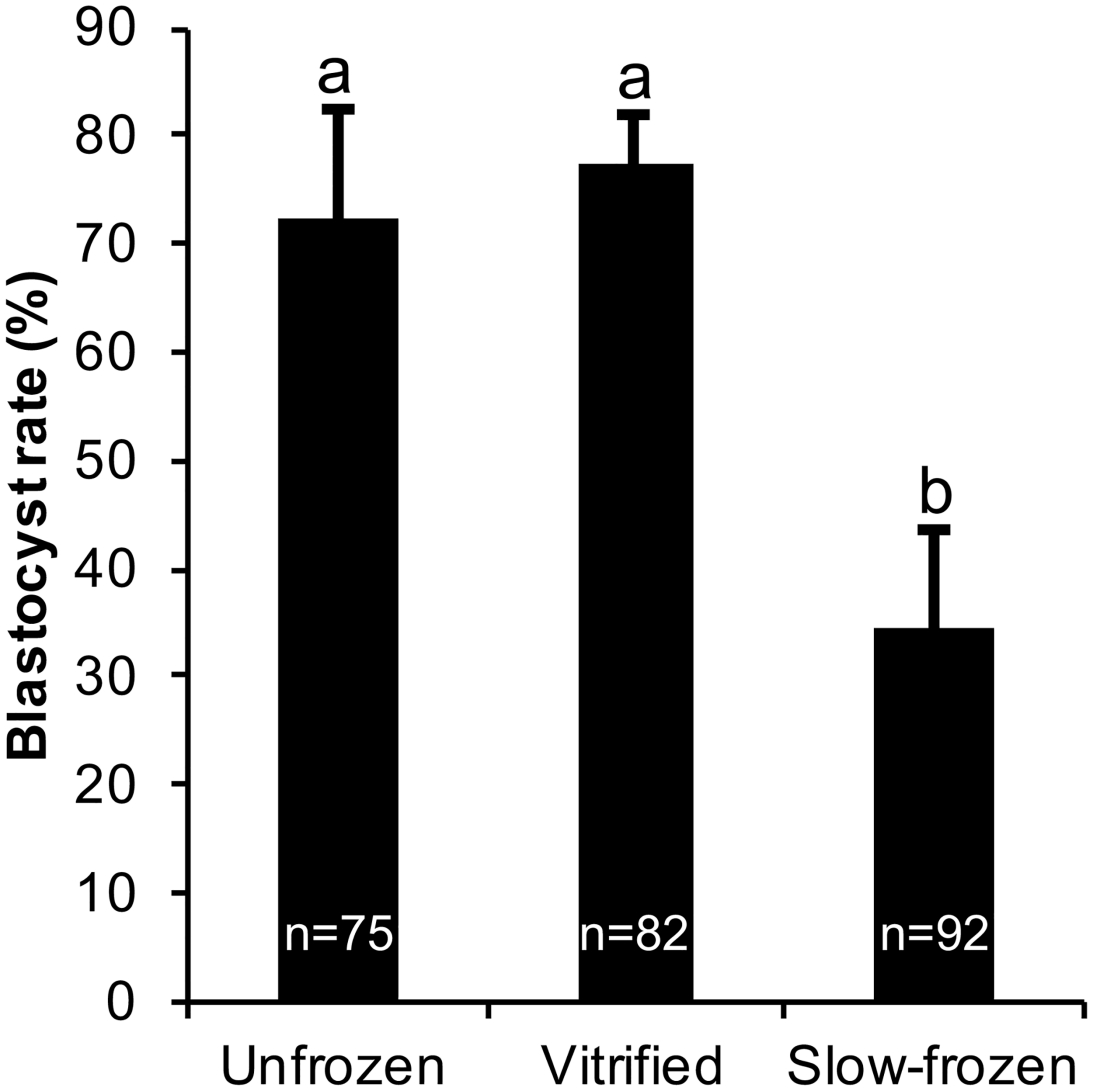
Blastocyst development rate (%; number of expanded blastocysts/number of morulae used per group) in unfrozen control, vitrified and slow-frozen bovine morulae. Each bar represents mean±SEM from five replicates. Different letters (a,b) on bars represent difference (*P* < 0.05) between groups.

The blastocyst development rate (number of expanded blastocysts/number of morulae used; %) did not differ between vitrification and control groups (*P* > 0.05). The slow freezing group had the lowest blastocyst development rate among all three groups (*P* < 0.05; Fig 1).

### Differential gene expression profile

Using FlexArray software, total 64 genes differentially expressed in blastocysts developed from vitrified compared to control morulae (fold change ≥ 2; *P* < 0.05); and 1 and 165 genes differentially expressed in blastocysts developed from slow-frozen compared to control morulae at fold change ≥ ± 2 and ≥ ± 1.5, respectively (*P* < 0.05; Table 2). *In silico* comparison revealed 75 genes differentially expressed in blastocysts developed from vitrified compared to slow-frozen morulae at fold change ≥ 2 (Table 2). Top 5 upregulated and 5 downregulated genes in blastocysts developed from vitrified vs. control morulae, slow-frozen vs. control morulae, and vitrified vs. slow-frozen morulae, are presented in Table 3.

**Table 2:** Upregulated and downregulated transcripts in blastocysts developed from vitrified and slow-frozen vs. unfrozen control (reference) morulae and in vitrified vs. slow-frozen (reference) morulae (*P* < 0.05). Transcripts with known functions and novel transcripts are listed separately.

Treatments	Fold change	Upregulated transcripts		Downregulated transcripts	
		Known	Novel	Known	Novel
**Vitrified vs. control**	≥±2	7	13	33	11
**Slow-frozen vs. control**	≥±2	0	0	1	0
**Slow-frozen vs. control**	≥±1.5	35	67	49	14
**Vitrified vs. slow-frozen**	≥±2	10	15	30	20

**Table 3:** Top five upregulated and downregulated genes detected by FlexArray^®^ analysis in blastocysts developed from vitrified vs. unfrozen control (reference) morulae, slow-frozen vs. unfrozen control (reference) morulae, vitrified vs, slow-frozen (reference) morulae.

Genes	Description	Fold change	*P* value
**Vitrified vs. control**			
*WBP5*	WW domain binding protein 5	2.148	1.49 X10^-05^
*TKTL1*	Transketolase 1	2.119	8.63 X10^-04^
*HS3ST5*	Heparan sulpfate (glucosamine)-O- sulphotransferase 5	2.113	6.68 X10^-04^
*TRIM64/TRIM64B*	Tripartite motif 64-B	2.100	1.33 X10^-05^
*COL9A2*	Collagen, type IX alpha 2	2.078	5.82 X10^-05^
*CYP11A1*	Cytochrome P450 family 11 subfamily A polypeptide 1	-3.971	3.43X10^-04^
*CCL17*	Chemokine (C-C motif) ligand 17	-2.946	4.39 X10^-04^
*FADS2*	Fatty acid desaturase 2	-2.897	1.54 X10^-04^
*HEBP2*	Heme binding protein 2	-2.817	5.48 X10^-05^
*KRT19*	Keratin 19	-2.784	2.36 X10^-03^
**Slow-frozen vs. control**			
*PLA2R1*	Phospholipase A 2 recepetor-1	1.983	3.99 X10^-03^
*DCLK2*	Doublecortin-like kinase-2	1.829	2.56 X10^-02^
*FBXO32*	F-box protein 32	1.774	8.90 X10^-04^
*SMPD3*	Sphingomyelin phosphodiestrase 3, neutral	1.772	7.48 X10^-03^
*ZMYM6*	Zinc finger, MYM-type 6	1.772	2.48 X10^-02^
*SPP1*	Secreted phosphoprotein 1	-2.197	1.14X10^-03^
*VIM*	Vimentin	-1.959	6.0 X10^-03^
*PAH*	Phenylalanine hydroxylase	-1.928	9.33 X10^-03^
*TBX18*	T-box 18	-1.886	1.07 X10^-02^
*TXNL4*	Thioredoxin like 4A	-1.857	4.10X10^-03^
**Vitrified vs. slow-frozen**			
*SDS*	Serine dehydratase	2.482	1.24 X10^-02^
*SERPINA5*	Serpin peptidase inhibitor, clade A (alpha 1, antiproteinase, antitrypsin), member 5	2.469	5.71 X10^-04^
*AGXT2L1*	Alanine-glyoxylate aminotransferase 2-like 1	2.326	2.48 X10^-03^
*LYZ3*	Lysozyme 3	2.284	2.10 X10^-03^
*GLDC*	Glycine dehydrogenase (decarboxylating)	2.161	2.64 X10^-03^
*NAGK*	N-acetylglucosamine kinase	-3.861	4.83X10^-02^
*CYP11A1*	Cytochrome P450, family 11, subfamily A, polypeptide 1	-3.326	7.89 X10^-04^
*CCL17*	Chemokine (C-C motif) ligand 17	-2.738	1.92 X10^-03^
*HEBP2*	Heme binding protein 2	-2.631	1.95 X10^-03^
*CLDN23*	Claudin 23	-2.455	9.85X10^-04^

### Upstream regulators

To understand broader implications of gene expression changes in blastocysts developed from vitrified and slow-frozen morulae, a list of potentially “inhibited” or “activated” upstream regulators was generated using IPA software. The analysis was based on the activation score ≥ ± 2 and *P* < 0.05. Activation score represents the direction of change for the function. Compared to blastocysts developed from control morulae, blastocysts developed from vitrified morulae had two “inhibited” upstream regulators i.e. *NFKB* and *Tretinoin* (Table 4). To provide a better picture, other upstream regulators are also listed in Table 4. Three upstream regulators (*NFKB*, *Tretinoin* and *EGF*) were common between blastocysts developed form vitrified vs. control morulae and vitrified vs. slow-frozen morulae.

**Table 4:** Upstream regulators predicted by IPA software to be inhibited/downregulated based on differential expression of target molecules identified in blastocysts developed from vitrified morulae vs. unfrozen control (reference) and vs. slow-frozen morulae (reference).

*Upstream regulator*	*Molecule type*	*Predicted activation state*	*Activation (z) score*	*P value*
***Vitrified vs*. *control***				
*NFKB*	Complex	Inhibited	*-2*.*165*	*1*.*55E-04*
*Tretinoin*	Chemical endogenous mammalian	Inhibited	*-2*.*124*	*1*.*68E-02*
*CEBPB*	Transcription regulator		*-1*.*972*	*2*.*57E-03*
*IGF1R*	Transmembrane receptor		*-1*.*969*	*2*.*08E-05*
*EGF*	Growth factor		*-1*.*831*	*8*.*30E-05*
*IFNG*	Cytokine		*-1*.*731*	*2*.*92E-04*
*TGFB1*	Growth factor		*-1*.*546*	*2*.*88E-03*
***Vitrified vs*. *slow-frozen***				
*NFKB*	Complex		*-1*.*921*	*1*.*18E-02*
*Tretinoin*	Chemical endogenous mammalian		*-1*.*886*	*1*.*89E-01*
*Beta -estradiol*	Chemical endogenous mammalian		*-1*.*561*	*9*.*33E-04*
*EGF*	Growth factor		*-1*.*194*	*8*.*00E-03*
*FGF2*	Growth factor		*-1*.*181*	*2*.*35E-03*
*IFNG*	Cytokine		*-1*.*137*	*4*.*80E-04*
*TNF*	Cytokine		*-1*.*043*	*1*.*03E-02*

### Functional annotation and pathway analysis

**Fig 2:**
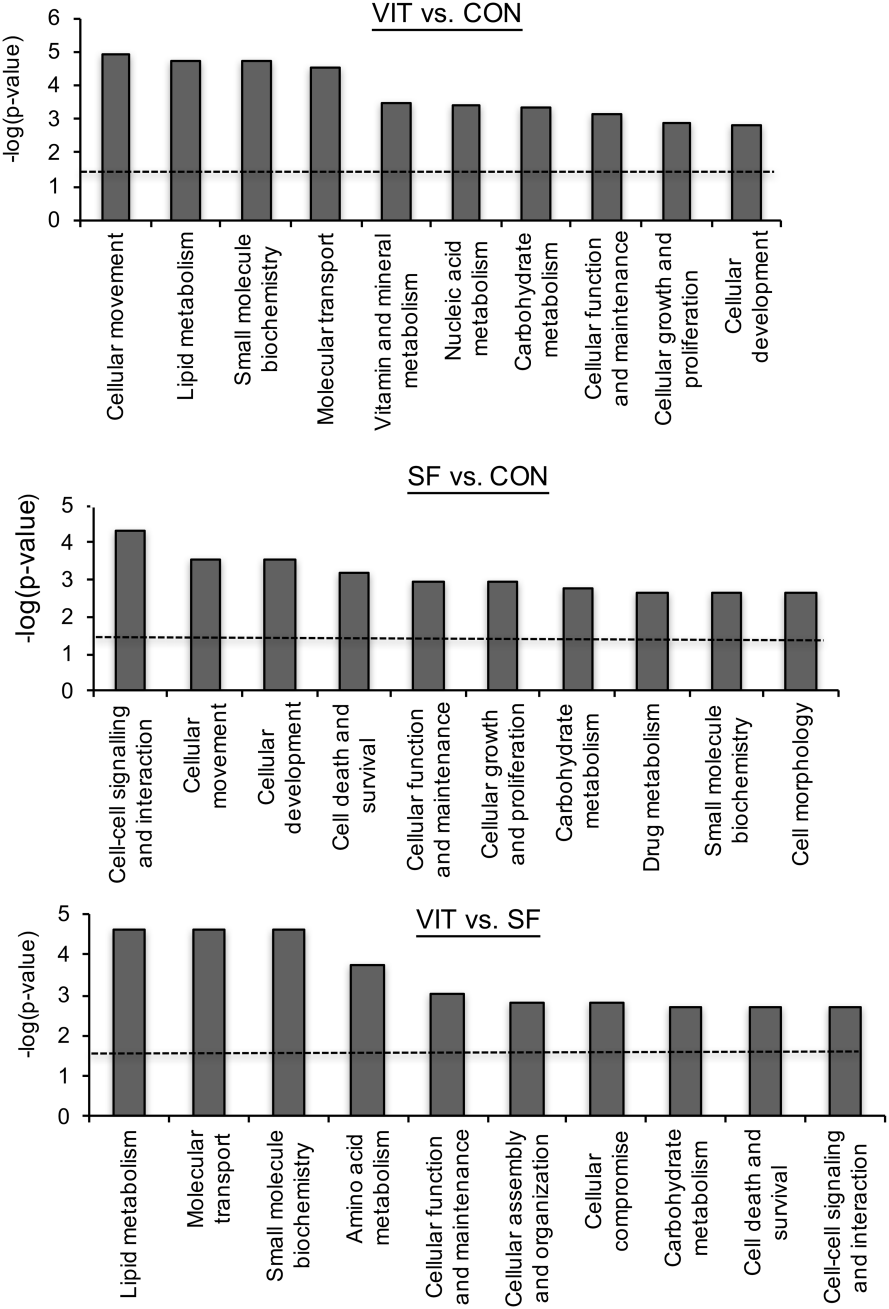
Functional analysis of differential gene expression in IVP bovine embryos based on–log (*P*-value) obtained with Ingenuity^®^ Pathway Analysis software. Higher log values relate to higher significance of the functions. Top 10 cellular and molecular functions in each comparison are illustrated. Taller bars are more significant than shorter bars and the dotted line represents the cut-off value for *P* ≤ 0.05, -log-value = 1.3. Abbreviations: CON–control; SF–slow freezing; VIT–vitrification.

**Fig 3:**
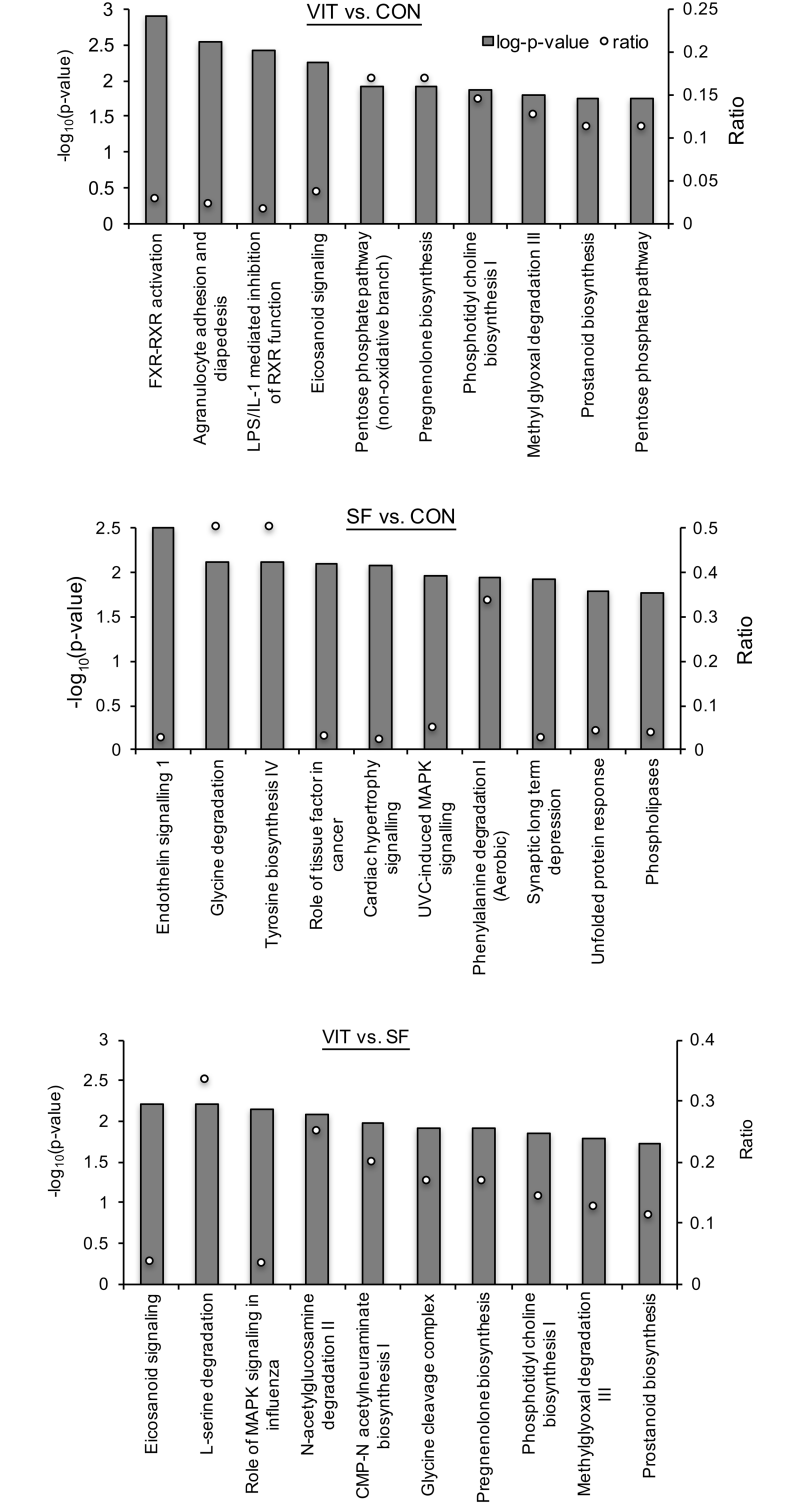
Canonical pathway analysis of gene expression in blastocysts developed from vitrified and slow-frozen vs. control morulae, and blastocysts developed from vitrified vs. slow-frozen morulae. Score ratio (open circles) depicts the number of genes affected in the treatment versus the total number of genes involved in the pathway (y-axis on right side of each figure).

**Fig 4:**
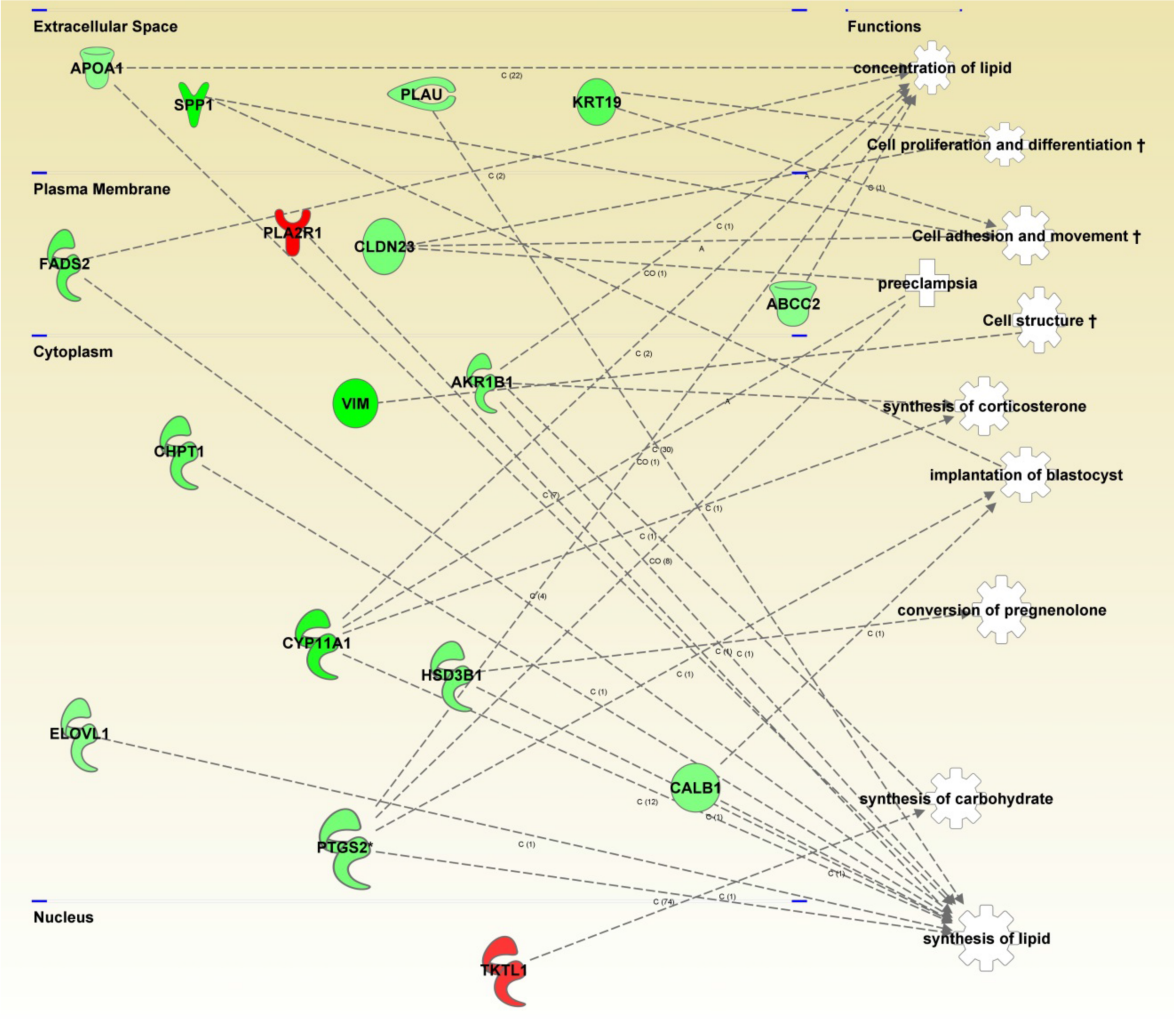
Functional network of differentially expressed genes in bovine blastocysts following vitrification at morula stage. All genes involved in this network are part of the matrix-remodeling network. Genes are arranged horizontally in four cell compartments (nucleus, cytoplasm, plasma membrane and extracellular space), based on subcellular location of their gene products. The differences in color intensity of molecules show the degree of up- (red) and down- (green) regulation. The relationship lines between molecules and functions are supported by at least one reference derived from the literature, textbooks, and/or canonical pathways stored in Ingenuity^®^ Knowledge Base.

The differentially expressed genes from the FlexArray analysis for each treatment comparison were uploaded in IPA software and analyzed to determine the affected cellular, molecular, physiological and disease-related pathways. Top 10 affected pathways in blastocysts developed from vitrified and slow-frozen morulae as compared with blastocysts developed from control morulae are presented in Fig 2. Among these, cellular movement, small molecule biochemistry, carbohydrate metabolism, cellular function and maintenance, cellular growth and proliferation, and cellular development pathways were common between blastocysts developed from vitrified and slow-frozen morulae. Similarly, 10 pathways affected in blastocysts developed from vitrified as compared with slow-frozen morulae are presented in Fig 2. Genes involved in well-known canonical pathways were also examined, using the IPA software and top 10 affected canonical pathways in vitrified vs. control, slow-frozen vs. control and vitrified vs. slow-frozen are presented in Fig 3. A network of top differentially expressed genes (*P* < 0.05; fold change ≥ ± 2) in blastocysts developed from vitrified as compared with control morulae is presented in Fig 4.

### Quantitative real-time PCR

**Fig 5:**
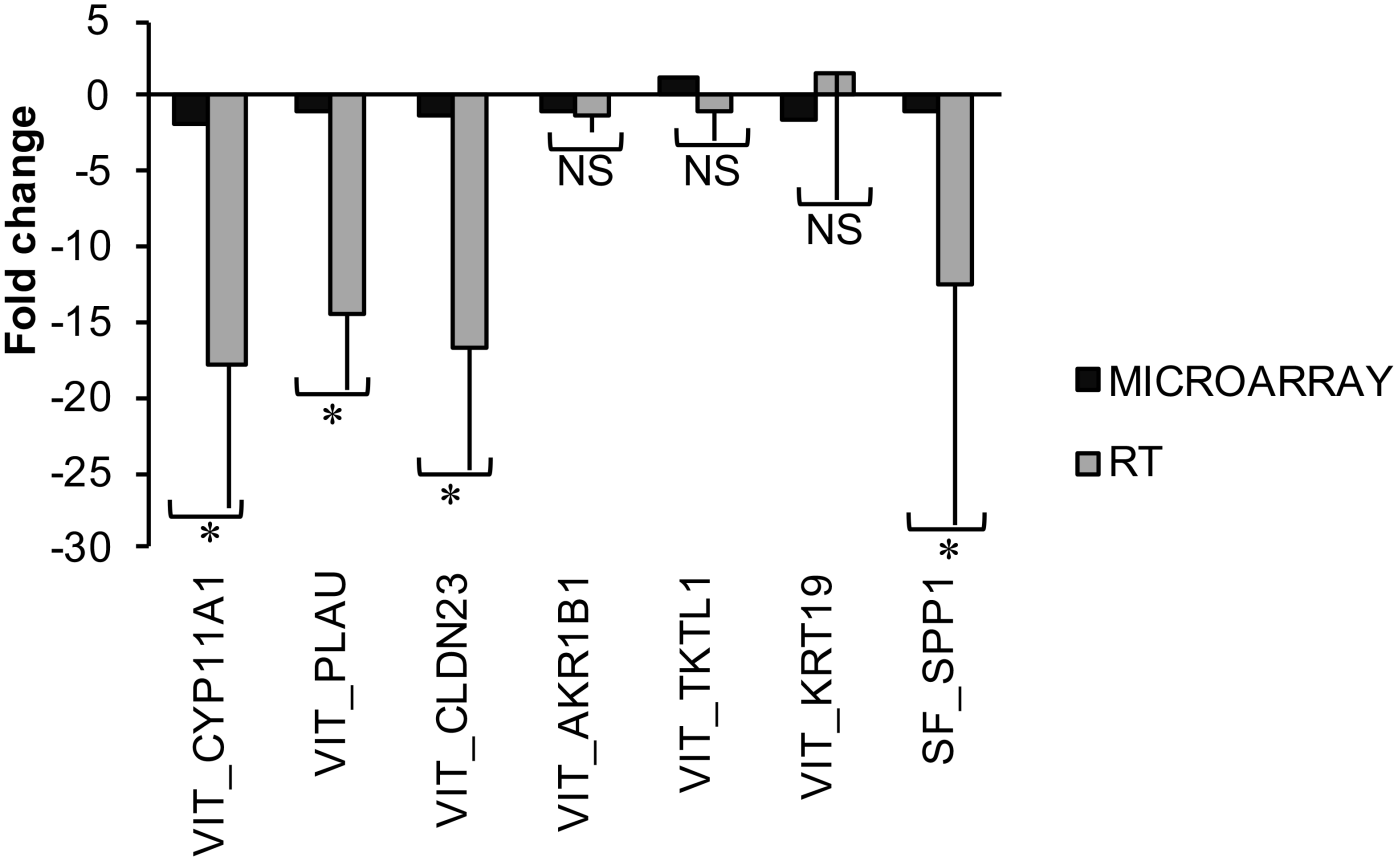
Quantification (fold-change; mean±SEM) of mRNA profiles of 7 genes in *in vitro* produced bovine blastocysts after cryopreservation treatment [Vitrification (VIT) vs. control and slow freezing (SF) vs. control] using qRT-PCR and microarray analyses (n = 3 replicates per morula group). Black bars represent the differential level of expression of transcripts detected in the microarray analysis, while light grey bars represent the differential level of expression of the same transcripts obtained by qRT-PCR analysis. Asterisks (*) represent difference between gene expressions determined by microarray and qRT-PCR (*P* ≤ 0.01) analyses. NS = nonsignificant.

Based on microarray data and function analysis, 6 differentially expressed genes (*AKR1B1*, *CLDN23*, *CYP11A1*, *KRT19*, *PLAU and TKTL1*) from vitrification group and 1 gene (*SPP1*) from slow freezing group were selected for validation with qRT-PCR. After quantification in three independent biological replicates from treatment (vitrification and slow freezing) and control groups, differential expression was validated in 7 genes (90% confidence level; P ≤ 0.01; Fig 5).

## Discussion

This is the first report, to our knowledge, on the comparison of differential gene expression in bovine blastocysts following vitrification and slow freezing at morula stage. In this study, vitrification of bovine morulae demonstrated better survival rate than slow freezing. However, this is the first report on the poor quality of surviving blastocysts developed from vitrified morulae at transcriptome level comparing with blastocysts developed from control morulae. Microarray data revealed that blastocysts developed from vitrified morulae may have impaired implantation and placentation in uterus. On the other side, the majority of morulae did not survive slow freezing method but those which survived to the blastocyst stage showed a similar transcriptome compared to blastocysts developed from control morulae.

In the present study, vitrification at morula stage affected the lipid metabolism and excretion mechanisms in *in vitro* produced bovine blastocysts. During slow freezing, the fluidic lipid portion of cell membrane changes into gel phase called ‘lipid phase transition’ [38]. In vitrification, embryos are exposed to the permeating cryoprotectants in high concentration and undergo ultra-fast cooling rate by direct plunging in liquid nitrogen [39]. In this procedure, embryos turn into glass-like solid phase, avoiding intracellular ice formation, as confirmed by synchrotron x-ray diffraction method [9]. However, vitrification causes irreversible damage to cell membranes in bovine embryos [40]. Membrane phospholipids [arachidonic acid and polyunsaturated fatty acids (PUFA)] are source for steroid metabolism. The current study revealed the downregulation of genes involved in steroid biosynthesis, pregnenolone biosynthesis and eicosanoid signaling (cytochrome P450 subunit 11 type A 1 (*CYP11A1*), 3-beta hydroxy steroid dehydrogenase delta-isomerase type 1 (*HSD3B1*), ATP-binding cassette subfamily C-2 (*ABCC2*) and prostaglandin synthase 2 (*PTGS2*) / cyclooxygenase 2 (*COX2*) in blastocysts developed from vitrified morulae. Earlier, the genes involved in purine metabolism and sphingolipid metabolism were upregulated in vitrified blastocysts [23]. This difference in gene regulation could be due to the difference in embryonic stage (morula vs. blastocyst) at which vitrification was done. The lipid metabolism genes (*CYP11A1*, *HSD3B1* and *APOA1*), involved in retinoids and their receptor (*FXR/RXR*) pathway were also downregulated in this study. *FXR/RXR* are expressed in inner cell mass (ICM) and trophectoderm (TE) cells and enhance blastocyst development and hatching in sheep and cattle [41,42].

In this study, *CYP11A1*, *HSD3B1*, *PTGS2* and aldo-keto reductases family 1 B1 (*AKR1B1*) genes were downregulated. These genes are involved in steroid metabolism and are important for embryo implantation and placentation [43,44]. The impaired gene expression of *CYP11A1* leads to inefficient lipid metabolism and defective placentation which are hallmarks of transcriptional deregulations in pre-eclamptic conditions [45]. Therefore, it is suggested that vitrification downregulates the genes involving implantation of bovine blastocysts. This hypothesis will be further discussed in subsequent sections along with other genes and pathways.

Vitrification is known for cytotoxicity associated with high concentration of the permeating cryoprotectants (DMSO and EG). This study demonstrated the downregulation of genes associated with cellular uptake and efflux of cholesterol and fatty acids [apolipoprotein type A1 (*APOA1*) and *ABCC2*] from the external media and are actively involved in cellular detoxification through waste disposal (detoxification) during oxidative stress [46–48]. This is an important mechanism for the survival of semi-independent pre-implantation embryos. Similarly, *AKR1B1* also performs detoxification that protects cells against lipid peroxidation products and toxic carbonyl-compounds produced during cell metabolism under stressful conditions [49].

The use of serum and/or bovine serum albumin in *in vitro culture* media renders bovine embryos susceptible to cryodamage due to increased cytoplasmic lipid content [50,51]. The downregulation of lipid metabolism and external uptake in stressful conditions may be responsible for better survival of vitrified than slow-frozen morulae to blastocyst stage. These findings support “quiet embryo” hypothesis i.e. embryo with relatively low metabolic activity survive better [52]. The addition of phenazine ethosulfate, a metabolic inhibitor for fatty acid synthesis, reduced lipid accumulation and increased blastocyst re-expansion after vitrification [53].

An important aspect of bovine embryo development is the blastocyst formation from morula stage which involves differentiation of blastomeres into ICM and TE cells. Claudin family and actinγ2 (*ACTG2*) are involved in formation of tight cell junctions in placental development [54,55]. These tight junctions prevent leakage of fluid during blastocoel formation, and support blastocyst expansion and hatching processes [56]. The downregulation of claudin and actin genes led us to develop a notion that vitrification delays the hatching of blastocysts. This supported previous findings that vitrification caused downregulation of tight junction and cell adhesion (tight junction protein and desmocollin2 genes in bovine blastocysts [28].

Cytokines (*IFNT* and *IFNG*) and growth factors (*EGF*, *FGF*, *TGFB* and *IGFBPB*) present in uterus, involved in cell growth, proliferation and differentiation, are important for transformation of morula to blastocyst and subsequent hatching. IFNT and IFNG play major roles in embryo signaling, maternal recognition of pregnancy, immune regulation and establishment of pregnancy [57,58]. IFNG secreted by the implanting embryos, inhibits the production of prostaglandin synthase (PGS) in bovine [59]. Interestingly, the expression of *CALB1*, *PTGS2*, *PLAU*, *KRT19* and *CYP11A1* genes was downregulated in blastocysts developed from vitrified morulae in this study. Another study failed to detect such change in IFN expression levels [28]. In the present study, keratin family (*KRT19*) and urokinase-based plasminogen activator (*PLAU*) genes were also downregulated in blastocysts developed from vitrified morulae. The PLAU is a key player in implantation and its downregulation has been associated with resorbed embryos in bovine [60]. Taken together, the downregulation of keratin family and PLAU along with predicted decrease in upstream regulator IFNG suggests the possible impairment in early embryo recognition and implantation process in vitrified embryos.

In the current study, IPA analysis of differentially expressed genes indicated apoptosis (*NFKB*, *CYP11A1*, *AKR1B1*, *CALB1* and *PLAU*) and necrosis (*TGFB*, *IFNG*, *PLAU*, *KRT19*, *CYP11A1*, *ABCC2*, and *CCL17*) pathways in blastocysts developed from vitrified morulae. Necrosis is a large scale cellular damage associated with membrane damage, nuclear disintegration and cellular swelling, thus may affect whole embryo survival [61]. Apoptosis, a physiological process, is usually associated with single cell damage i.e. cytoplasmic shrinkage, chromatin condensation and DNA damage leaving adjacent cells intact. Apoptosis of individual blastomere is a part of strategy of embryo survival under stressful circumstances [62]. This seems to be true for greater survival rate in vitrified morulae than slow-frozen.

Interestingly, the surviving blastocysts developed from slow-frozen morulae showed fewer changes in gene expression comparing with blastocysts developed from control morulae but differed significantly comparing with blastocysts developed from vitrified morulae. The upregulation of cell structure and morphology genes microtubule polymerization, doublecortin-like kinase 2 (*DCLK2*) and zinc finger MYM 6 (*ZMYM6*) compared to control group may be the compensatory mechanisms for cell structure damage. Vimentin (*VIM*) was also downregulated in blastocysts developed from slow-frozen morulae. VIM encodes a protein member of cellular intermediate filaments known to enhance cell elasticity, capacity to adapt stress and thus is important for normal bovine embryo development [63]. Other upregulated genes in slow-frozen blastocysts related to membrane lipid metabolizing enzymes i.e. phospholipase A2 recepetor 1 (*PLA2R1*) and sphingomyelin phosphodiestrase 3 (*SMPD3*) depict the utilization of embryo’s internal resources for metabolism. These changes point towards the viability and better quality of blastocysts developed from slow-frozen which survived the transition from morula to blastocyst after warming.

Vitrification is becoming popular under field conditions due to high embryo survival rates as well as the ease of technique [10]. Their study demonstrated higher morula to blastocyst rate in vitrified blastocysts (77%) than slow-frozen blastocysts (34%). Similar results were obtained in the present and earlier studies on bovine embryos [10,64,65]. Interestingly, *in silico* comparison between blastocysts developed from vitrified and slow-frozen morulae revealed similar changes in gene expression as between blastocysts from vitrified and control morulae. Similar pathways like lipid metabolism and cell movement and adhesion were affected. Inspite of better survival, a similar pregnancy rate (~45%) was observed after transfer of vitrified and slow-frozen bovine embryos, under field conditions [12]. Studies conducted on rabbit morulae following vitrification demonstrated impaired trophoblast proliferation and differentiation, retarded fetal development and altered gene expression (*ANXA3*, *EGFLAM* and *TNAIP6*) compared to slow-frozen blastocysts [66,67]. Based on discussion in previous sections and considering the field pregnancy data in cattle, we anticipate that vitrified blastocysts compared to slow-frozen blastocysts have higher failure rate during maternal recognition of pregnancy and pre-implantation, resulting in similar pregnancy rates by 30-days of gestation. This hypothesis needs to be tested further.

Bovine embryos between compact morula- and blastocyst-stage, suitable for non-surgical embryo transfer, are frozen with great success [13]. Morula/early blastocyst is a favourite stage for cryopreservation to avoid embryo manipulation like blastocoel collapse in the expanded blastocyst [68]. In this study, compact morulae were cryopreserved so that post-warm transcriptomic changes could be expressed at later (blastocyst) stage. This study represented transcriptomic changes in bovine embryos cryopreserved at morula stage. It is anticipated that transcriptomic changes of bovine embryos may be different if cryopreserved at the expanded blastocyst stage.

## Conclusions

Blastocysts developed from vitrified morulae showed downregulation of genes involved in lipid metabolism, cell differentiation and cell adhesion leading to impaired implantation. Although the survival rate of blastocysts developed from slow-frozen morulae was poor, the intensity of changes in gene expression was low comparing with blastocysts developed from unfrozen control morulae. Also, gene expression changes between blastocysts developed from vitrified vs. slow-frozen morulae were similar as between blastocysts developed from vitrified vs. control morulae. Generally, the cryosurvival of morulae is assessed at blastocyst, expanded blastocyst and/or hatched blastocyst stages. It will be important to study the developmental competence of cryopreserved embryos beyond blastocyst stage, like implantation, placentation and actual pregnancy.

## References

[1] Leibo SP. Cryopreservation of oocytes and embryos: optimization by theoretical versus empirical analysis. Theriogenology. 2008;69: 37–47. doi: 10.1016/j.theriogenology.2007.10.006 18023472 10.1016/j.theriogenology.2007.10.006

[2] Vajta G, Kuwayama M. Improving cryopreservation systems. Theriogenology. 2006;65: 236–244. doi: 10.1016/j.theriogenology.2005.09.026 16289262 10.1016/j.theriogenology.2005.09.026

[3] Mazur P. Cryobiology: the freezing of biological systems. Science. 1970;168: 939–949. 5462399 10.1126/science.168.3934.939

[4] Wilmut I. The low temperature preservation of mammalian embryos. J Reprod Fertil. 1972;31: 513–514. 4120076 10.1530/jrf.0.0310513

[5] Pollard JW, Leibo SP. Chilling Sensitivity of Mammalian embryos. Theriogenology. 1994;41: 101–107.

[6] Leibo SP, Pool TB. The principal variables of cryopreservation: solutions, temperatures, and rate changes. Fertil Steril. 2011;96: 269–276. doi: 10.1016/j.fertnstert.2011.06.065 21782053 10.1016/j.fertnstert.2011.06.065

[7] Kuwayama M. Highly efficient vitrification for cryopreservation of human oocytes and embryos: the Cryotop method. Theriogenology. 2007;67: 73–80. doi: 10.1016/j.theriogenology.2006.09.014 17055564 10.1016/j.theriogenology.2006.09.014

[8] Rall WF, Fahy GM. Ice-free cryopreservation of mouse embryos at -196°C by vitrification. Nature. 1985;313: 573–575. 3969158 10.1038/313573a0

[9] Anzar M, Grochulski P, Bonnet B. Synchrotron X-ray diffraction to detect glass or ice formation in the vitrified bovine cumulus-oocyte complexes and morulae. PLoS One. 2014;9: e114801 doi: 10.1371/journal.pone.0114801 25536435 10.1371/journal.pone.0114801 PMC4275205

[10] Vajta G, Nagy ZP. Are programmable freezers still needed in the embryo laboratory? Review on vitrification. Reprod Biomed Online. 2006;12: 779–796. 16792858 10.1016/s1472-6483(10)61091-7

[11] Nedambale TL, Dinnyes A, Groen W, Dobrinsky JR, Tian XC, Yang X. Comparison on in vitro fertilized bovine embryos cultured in KSOM or SOF and cryopreserved by slow freezing or vitrification. Theriogenology. 2004;62: 437–449. doi: 10.1016/j.theriogenology.2003.10.020 15226000 10.1016/j.theriogenology.2003.10.020

[12] van Wagtendonk-de Leeuw AM, den Daas JH, Rall WF. Field trial to compare pregnancy rates of bovine embryo cryopreservation methods: vitrification and one-step dilution versus slow freezing and three-step dilution. Theriogenology. 1997;48: 1071–1084. 16728196 10.1016/s0093-691x(97)00340-3

[13] Bondioli K. Cryopreservation of Bovine Embryos In: Hopper R, editor. Bovine Reproduction. Hoboken, NJ, USA: John Wiley & Sons, Inc; 2014 pp. 718–722.

[14] Lehn-Jensen H, Rall WF. Cryomicroscopic observations of cattle embryos during freezing and thawing. Theriogenology. 1983;19: 263–277. 16725794 10.1016/0093-691x(83)90013-4

[15] Leibo SP, McGrath JJ, Cravalho EG. Microscopic observation of intracellular ice formation in unfertilized mouse ova as a function of cooling rate. Cryobiology. 1978;15: 257–271. 710156 10.1016/0011-2240(78)90036-6

[16] Rekik W, Dufort I, Sirard MA. Analysis of the gene expression pattern of bovine blastocysts at three stages of development. Mol Reprod Dev. 2011;78: 226–240. doi: 10.1002/mrd.21286 21509852 10.1002/mrd.21286

[17] Vigneault C, McGraw S, Massicotte L, Sirard MA. Transcription factor expression patterns in bovine in vitro-derived embryos prior to maternal-zygotic transition. Biol Reprod. 2004;70: 1701–1709. doi: 10.1095/biolreprod.103.022970 14960490 10.1095/biolreprod.103.022970

[18] Yao J, Ren X, Ireland JJ, Coussens PM, Smith TP, Smith GW. Generation of a bovine oocyte cDNA library and microarray: resources for identification of genes important for follicular development and early embryogenesis. Physiol Genomics. 2004;19: 84–92. doi: 10.1152/physiolgenomics.00123.2004 15375196 10.1152/physiolgenomics.00123.2004

[19] Gendelman M, Roth Z. Seasonal effect on germinal vesicle-stage bovine oocytes is further expressed by alterations in transcript levels in the developing embryos associated with reduced developmental competence. Biol Reprod. 2012;86: 1–9. 10.1095/biolreprod.111.092882 21957191

[20] Carter F, Rings F, Mamo S, Holker M, Kuzmany A, Besenfelder U, et al Effect of elevated circulating progesterone concentration on bovine blastocyst development and global transcriptome following endoscopic transfer of in vitro produced embryos to the bovine oviduct. Biol Reprod. 2010;83: 707–719. doi: 10.1095/biolreprod.109.082354 20631399 10.1095/biolreprod.109.082354

[21] Gilbert I, Robert C, Vigneault C, Blondin P, Sirard MA. Impact of the LH surge on granulosa cell transcript levels as markers of oocyte developmental competence in cattle. Reproduction. 2012;143: 735–747. doi: 10.1530/REP-11-0460 22457433 10.1530/REP-11-0460

[22] Dias FC, Khan MI, Sirard MA, Adams GP, Singh J. Differential gene expression of granulosa cells after ovarian superstimulation in beef cattle. Reproduction. 2013;146: 181–191. doi: 10.1530/REP-13-0114 23740080 10.1530/REP-13-0114

[23] Aksu DA, Agca C, Aksu S, Bagis H, Akkoc T, Caputcu AT, et al Gene expression profiles of vitrified in vitro- and in vivo-derived bovine blastocysts. Mol Reprod Dev. 2012;79: 613–625. doi: 10.1002/mrd.22068 22778065 10.1002/mrd.22068

[24] Dhali A, Anchamparuthy VM, Butler SP, Pearson RE, Mullarky IK, Gwazdauskas FC. Gene expression and development of mouse zygotes following droplet vitrification. Theriogenology. 2007;68: 1292–1298. doi: 10.1016/j.theriogenology.2007.08.030 17915304 10.1016/j.theriogenology.2007.08.030

[25] Morato R, Izquierdo D, Paramio MT, Mogas T. Survival and apoptosis rates after vitrification in cryotop devices of in vitro-produced calf and cow blastocysts at different developmental stages. Reprod Fertil Dev. 2010;22: 1141–1147. doi: 10.1071/RD10013 20797352 10.1071/RD10013

[26] Li L, Zhang X, Zhao L, Xia X, Wang W. Comparison of DNA apoptosis in mouse and human blastocysts after vitrification and slow freezing. Mol Reprod Dev. 2012;79: 229–236. doi: 10.1002/mrd.22018 22213487 10.1002/mrd.22018

[27] Martino NA, Dell'aquila ME, Cardone RA, Somoskoi B, Lacalandra GM, Cseh S. Vitrification preserves chromatin integrity, bioenergy potential and oxidative parameters in mouse embryos. Reprod Biol Endocrinol. 2013;11: 27 doi: 10.1186/1477-7827-11-27 23552480 10.1186/1477-7827-11-27 PMC3652727

[28] Stinshoff H, Wilkening S, Hanstedt A, Bruning K, Wrenzycki C. Cryopreservation affects the quality of in vitro produced bovine embryos at the molecular level. Theriogenology. 2011;76: 1433–1441. doi: 10.1016/j.theriogenology.2011.06.013 21835456 10.1016/j.theriogenology.2011.06.013

[29] de Loos F, van Vliet C, van Maurik P, Kruip TA. Morphology of immature bovine oocytes. Gamete Res. 1989;24: 197–204. doi: 10.1002/mrd.1120240207 2793058 10.1002/mrd.1120240207

[30] Parrish JJ, Krogenaes A, Susko-Parrish JL. Effect of bovine sperm separation by either swim-up or Percoll method on success of in vitro fertilization and early embryonic development. Theriogenology. 1995;44: 859–869. 16727781 10.1016/0093-691x(95)00271-9

[31] Brackett BG, Oliphant G. Capacitation of rabbit spermatozoa in vitro. Biol Reprod. 1975;12: 260–274. 1122333 10.1095/biolreprod12.2.260

[32] Rosenkrans CF Jr., Zeng GQ, MC GT, Schoff PK, First NL. Development of bovine embryos in vitro as affected by energy substrates. Biol Reprod. 1993;49: 459–462. 8399836 10.1095/biolreprod49.3.459

[33] Prentice JR, Singh J, Dochi O, Anzar M. Factors affecting nuclear maturation, cleavage and embryo development of vitrified bovine cumulus-oocyte complexes. Theriogenology. 2011;75: 602–609. doi: 10.1016/j.theriogenology.2010.09.027 21190729 10.1016/j.theriogenology.2010.09.027

[34] Carvalho RV, Del Campo MR, Palasz AT, Plante Y, Mapletoft RJ. Survival rates and sex ratio of bovine IVE embryos frozen at different developmental stages on day 7. Theriogenology. 1996;45: 489–498. 16727812 10.1016/0093-691x(95)00385-l

[35] Cagnone GL, Dufort I, Vigneault C, Sirard MA. Differential gene expression profile in bovine blastocysts resulting from hyperglycemia exposure during early cleavage stages. Biol Reprod. 2012;86: 50 doi: 10.1095/biolreprod.111.094391 22075474 10.1095/biolreprod.111.094391

[36] Robert C, Nieminen J, Dufort I, Gagne D, Grant JR, Cagnone G, et al Combining resources to obtain a comprehensive survey of the bovine embryo transcriptome through deep sequencing and microarrays. Mol Reprod Dev. 2011;78: 651–664. doi: 10.1002/mrd.21364 21812063 10.1002/mrd.21364

[37] Bolstad BM, Irizarry RA, Astrand M, Speed TP. A comparison of normalization methods for high density oligonucleotide array data based on variance and bias. Bioinformatics. 2003;19: 185–193. 12538238 10.1093/bioinformatics/19.2.185

[38] Leibo SP. Preservation of ova and embryos by freezing In: Brackett EG S G, Seidel SM, editor. New Technologies in Animal Breeding. New York: Academic press; 1981 pp. 127–139.

[39] Fahy GM, MacFarlane DR, Angell CA, Meryman HT. Vitrification as an approach to cryopreservation. Cryobiology. 1984;21: 407–426. 6467964 10.1016/0011-2240(84)90079-8

[40] Leao BC, Rocha-Frigoni NA, Cabral EC, Franco MF, Ferreira CR, Eberlin MN, et al Membrane lipid profile monitored by mass spectrometry detected differences between fresh and vitrified in vitro-produced bovine embryos. Zygote. 2014: 1–10. 25213102 10.1017/S0967199414000380

[41] Eberhardt DM, Will WA, Godkin JD. Retinol administration to superovulated ewes improves in vitro embryonic viability. Biol Reprod. 1999;60: 1483–1487. 10330109 10.1095/biolreprod60.6.1483

[42] Mohan M, Malayer JR, Geisert RD, Morgan GL. Expression of retinol-binding protein messenger RNA and retinoic acid receptors in preattachment bovine embryos. Mol Reprod Dev. 2001;60: 289–296. doi: 10.1002/mrd.1090 11599039 10.1002/mrd.1090

[43] Shemesh M, Izhar M, Pasmanik M, Shore LS. Regulation of steroidogenesis in the bovine placenta. J Physiol Pharmacol. 1992;43: 153–163. 1343967

[44] Breuiller-Fouche M, Leroy MJ, Dubois O, Reinaud P, Chissey A, Qi H, et al Differential expression of the enzymatic system controlling synthesis, metabolism, and transport of PGF2 alpha in human fetal membranes. Biol Reprod. 2010;83: 155–162. doi: 10.1095/biolreprod.109.080390 20357271 10.1095/biolreprod.109.080390

[45] Vaiman D, Calicchio R, Miralles F. Landscape of transcriptional deregulations in the preeclamptic placenta. PLoS One. 2013;8: e65498 doi: 10.1371/journal.pone.0065498 23785430 10.1371/journal.pone.0065498 PMC3681798

[46] Abumrad NA, Sfeir Z, Connelly MA, Coburn C. Lipid transporters: membrane transport systems for cholesterol and fatty acids. Curr Opin Clin Nutr Metab Care. 2000;3: 255–262. 10929670 10.1097/00075197-200007000-00003

[47] Rigotti A, Krieger M. Getting a handle on "good" cholesterol with the high-density lipoprotein receptor. N Engl J Med. 1999;341: 2011–2013. doi: 10.1056/NEJM199912233412612 10607822 10.1056/NEJM199912233412612

[48] Boisvert WA, Black AS, Curtiss LK. ApoA1 reduces free cholesterol accumulation in atherosclerotic lesions of ApoE-deficient mice transplanted with ApoE-expressing macrophages. Arterioscler Thromb Vasc Biol. 1999;19: 525–530. 10073953 10.1161/01.atv.19.3.525

[49] Sanchez-Gomez FJ, Diez-Dacal B, Garcia-Martin E, Agundez JA, Pajares MA, Perez-Sala D. Detoxifying enzymes at the cross-roads of inflammation, oxidative stress, and drug hypersensitivity: Role of glutathione transferase p1-1 and aldose reductase. Front Pharmacol. 2016;7: 237 doi: 10.3389/fphar.2016.00237 27540362 10.3389/fphar.2016.00237 PMC4973429

[50] Abe H, Yamashita S, Satoh T, Hoshi H. Accumulation of cytoplasmic lipid droplets in bovine embryos and cryotolerance of embryos developed in different culture systems using serum-free or serum-containing media. Mol Reprod Dev. 2002;61: 57–66. doi: 10.1002/mrd.1131 11774376 10.1002/mrd.1131

[51] Mucci N, Aller J, Kaiser GG, Hozbor F, Cabodevila J, Alberio RH. Effect of estrous cow serum during bovine embryo culture on blastocyst development and cryotolerance after slow freezing or vitrification. Theriogenology. 2006;65: 1551–1562. doi: 10.1016/j.theriogenology.2005.08.020 16229883 10.1016/j.theriogenology.2005.08.020

[52] Leese HJ. Quiet please, do not disturb: a hypothesis of embryo metabolism and viability. BioEssays. 2002;24: 845–849. doi: 10.1002/bies.10137 12210521 10.1002/bies.10137

[53] Sudano MJ, Paschoal DM, Rascado Tda S, Magalhaes LC, Crocomo LF, de Lima-Neto JF, et al Lipid content and apoptosis of in vitro-produced bovine embryos as determinants of susceptibility to vitrification. Theriogenology. 2011;75: 1211–1220. doi: 10.1016/j.theriogenology.2010.11.033 21247620 10.1016/j.theriogenology.2010.11.033

[54] Leach L, Lammiman MJ, Babawale MO, Hobson SA, Bromilou B, Lovat S, et al Molecular Organization of Tight and Adherens Junctions in the Human Placental Vascular Tree. Placenta. 2000;21: 547–557. doi: 10.1053/plac.2000.0541 10940205 10.1053/plac.2000.0541

[55] Turksen K, Troy TC. Claudin-6: a novel tight junction molecule is developmentally regulated in mouse embryonic epithelium. Dev Dyn. 2001;222: 292–300. doi: 10.1002/dvdy.1174 11668606 10.1002/dvdy.1174

[56] Watson AJ, Barcroft LC. Regulation of blastocyst formation. Front Biosci. 2001;6: D708–730. 11333210 10.2741/watson

[57] Charpigny G, Reinaud P, Huet JC, Guillomot M, Charlier M, Pernollet JC, et al High homology between a trophoblastic protein (trophoblastin) isolated from ovine embryo and alpha-interferons. FEBS Letters. 1988;228: 12–16. 3254170 10.1016/0014-5793(88)80574-x

[58] Sharkey A. Cytokines and implantation. Rev Reprod. 1998;3: 52–61. 9509989 10.1530/ror.0.0030052

[59] Godkin JD, Smith SE, Johnson RD, Dore JJ. The role of trophoblast interferons in the maintenance of early pregnancy in ruminants. Am J Reprod Immunol. 1997;37: 137–143. 9138447 10.1111/j.1600-0897.1997.tb00202.x

[60] El-Sayed A, Hoelker M, Rings F, Salilew D, Jennen D, Tholen E, et al Large-scale transcriptional analysis of bovine embryo biopsies in relation to pregnancy success after transfer to recipients. Physiol Genomics. 2006;28: 84–96. doi: 10.1152/physiolgenomics.00111.2006 17018689 10.1152/physiolgenomics.00111.2006

[61] Proskuryakov S, Konoplyannikov A, Gabai LV. Necrosis: a specific form of programmed cell death? Exp Cell Res. 2003;283: 1–16. 12565815 10.1016/s0014-4827(02)00027-7

[62] Antunes G, Chaveiro A, Santos P, Marques A, Jin HS, Moreira da Silva F. Influence of apoptosis in bovine embryo's development. Reprod Domest Anim. 2010;45: 26–32. doi: 10.1111/j.1439-0531.2008.01131.x 19055557 10.1111/j.1439-0531.2008.01131.x

[63] Maddox-Hyttel P, Alexopoulos NI, Vajta G, Lewis I, Rogers P, Cann L, et al Immunohistochemical and ultrastructural characterization of the initial post-hatching development of bovine embryos. Reproduction. 2003;125: 607–623. 12683931

[64] Nedambale TL, Dinnyes A, Yang X, Tian XC. Bovine blastocyst development in vitro: timing, sex, and viability following vitrification. Biol Reprod. 2004;71: 1671–1676. doi: 10.1095/biolreprod.104.027987 15253921 10.1095/biolreprod.104.027987

[65] Gupta A, Singh J, Anzar M. Effect of cryopreservation technique and season on the survival of in vitro produced cattle embryos. Anim Reprod Sci. 2016;164: 162–168. doi: 10.1016/j.anireprosci.2015.11.026 26679433 10.1016/j.anireprosci.2015.11.026

[66] Vicente JS, Saenz-de-Juano MD, Jimenez-Trigos E, Viudes-de-Castro MP, Penaranda DS, Marco-Jimenez F. Rabbit morula vitrification reduces early foetal growth and increases losses throughout gestation. Cryobiology. 2013;67: 321–326. doi: 10.1016/j.cryobiol.2013.09.165 24080489 10.1016/j.cryobiol.2013.09.165

[67] Saenz-de-Juano MD, Marco-Jimenez F, Viudes-de-Castro MP, Lavara R, Vicente JS. Direct comparison of the effects of slow freezing and vitrification on late blastocyst gene expression, development, implantation and offspring of rabbit morulae. Reprod Domest Anim. 2014;49: 505–511. doi: 10.1111/rda.12320 24750498 10.1111/rda.12320

[68] Zander-Fox D, Lane M, Hamilton H. Slow freezing and vitrification of mouse morula and early blastocysts. J Assist Reprod Genet. 2013;30: 1091–1098. doi: 10.1007/s10815-013-0056-4 23888311 10.1007/s10815-013-0056-4 PMC3790113

